# Progress towards revealing the mechanism of herpesvirus capsid maturation and genome packaging

**DOI:** 10.1007/s13238-020-00716-8

**Published:** 2020-04-08

**Authors:** Zhihai Li, Xuekui Yu

**Affiliations:** 1 Cryo-Electron Microscopy Research Center, the CAS Key Laboratory of Receptor Research, Shanghai Institute of Materia Medica, Chinese Academy of Sciences, Shanghai, 201203 China; 2 University of Chinese Academy of Sciences, Beijing, 100049 China

Members in the family *Herpesviridae* share a common architecture: the outer envelope, the inner nucleocapsid and the in-between tegument. There are three types of capsids, A-, B- and C-capsids, which are matured from the procapsid. The procapsid is assembled in the host cell nucleus where the viral dsDNA genome is packaged. The viral genome is driven into the procapsid by the terminase through the portal, which locates at a unique vertex of the icosahedral capsid (Heming et al., 2017). The A-capsid is empty and is believed to be resulted from unsuccessfully packaging of viral genome. B-capsid contains the scaffold similar to that of the procapsid and is likely resulted from failed initiation of viral DNA packaging. C-capsid contains the full-length viral genome and is able to further mature into the infectious virion. With the advance of cryo-electron microscopy (cryo-EM), the high-resolution structures of herpesvirus capsid (Yu et al., 2017; Dai and Zhou, 2018; Dai et al., 2018; Wang et al., 2018; Yuan et al., 2018) and the unique portal-vertex (Gong et al., 2019; Liu et al., 2019) of the infectious virion have been determined recently. These results showed that the structures of the herpesvirus major capsid protein and the portal, as well as the interactions among them closely resemble that of the tailed DNA bacteriophage, indicating the herpesvirus shares a common mechanism of the capsid assembly and genome package with the bacteriophage (Yu et al., 2017; Dai and Zhou, 2018; Dai et al., 2018; Wang et al., 2018; Yuan et al., 2018; Gong et al., 2019; Liu et al., 2019; Chen et al., 2020). Although these achievements greatly advanced our understanding the structures and functions of herpesvirus capsid and portal, there are still questions regarding the herpesvirus capsid assembly and genome package: 1) what’s the working mechanism of the herpesvirus terminase? and 2) how is the viral capsid maturation coupled with the genome packaging? Two papers published back-to-back in this issue of *Protein* & *Cell* by Wang’s group shed light on these questions (Nan Wang, 2020; Yunxiang Yang, 2020).

The researchers determined the asymmetric structures of herpesvirus simplex virus 2 (HSV-2), B-, C- and virion-capsids by using a block-based reconstruction method (Wang et al., 2018; Yuan et al., 2018; Wang et al., 2019). A comparison of asymmetric reconstructions of the portal vertex of the three capsids reveals notable differences in the position and morphology of the portal and portal vertex associated tegument (PVAT). The portal in the B-capsid locates inwards by ~30 Å compared to the counterparts of C-and virion-capsids. In addition, the B-capsid portal is lack of the PVAT and is thus more accessible by the terminase, which may represent a ready-state for docking of terminase complexes. Given that the B-capsid is likely a product of unsuccessfully initiating of viral DNA package, the portal of B-capsid should somewhat resemble that of the procapsid, suggesting that the portal of procapsid could function as a DNA-packaging sensor to trigger the conformational changes of capsid during capsid maturation.

It is a long-time mystery over whether the terminase in dsDNA viruses is a pentameric rotation motor or a hexameric revolution motor (Aathavan et al., 2009; Moffitt et al., 2009; Schwartz et al., 2013; Hilbert et al., 2015; Guo et al., 2016). The first *in vitro*-assembled intact herpesvirus terminase complexes obtained by Wang’s group are predominantly in hexamer. The researchers then determined the atomic resolution structure of the hexameric terminase complex by using block-reconstruction method. The molecular rearrangement between the ATPase domain and nuclease domain in the terminase caused by ligand binding, as well as the big central channel of the hexameric motor (39 Å in diameter) observed by the researchers favor the previously proposed revolution model of viral DNA translocation and cleavage. Considering the fact that a majority region in the viral portal turret (the putative site for terminase complex anchoring) in mature capsids were determined to be disordered (Gong et al., 2019; Liu et al., 2019; Nan Wang, 2020), we cannot even exclude the possibility that the DNA packaging motors have the ability to transform between pentamer and hexamer during DNA packaging. It would be fabulous to see the *in situ* structure of the viral terminase, while it is indeed too challenging since that tremendous efforts of many investigators over two decades failed.
